# A Case of a Repeatedly Missed Aneurysm in a Patient With Third Nerve Palsy

**DOI:** 10.7759/cureus.114376

**Published:** 2026-08-11

**Authors:** Du Cheng, Tulsi Mehta, Sharanya Deshmukh, Evan Silverstein

**Affiliations:** 1 Ophthalmology, Virginia Commonwealth University Health Systems, Richmond, USA

**Keywords:** computed tomographic angiography, eyelid ptosis, oculomotor nerve (cn iii) palsy, posterior communicating artery (pcom), true posterior communicating artery aneurysm

## Abstract

Medically complex presentations may pose a diagnostic challenge for providers, highlighting the need for inter-team communication and independent imaging review. We report a single case in which the patient presented with cranial nerve III palsy from a posterior communicating artery (PCOM) aneurysm that was missed on three separate angiographies. The patient initially presented with an incomplete cranial nerve (CN) III palsy and had workup and treatment for giant cell arteritis (GCA). Three months later, she presented again with worsened, complete CN III palsy. Upon independent review of CT and MRI angiography by the ophthalmology and neuro-radiology team, an 8 mm aneurysm of the right internal carotid artery (ICA) adjacent to the posterior communicating artery (PCOM) (initially missed) was identified. The patient was treated with percutaneous coil embolization, which resulted in partial recovery of her CN III function. This case demonstrates the importance of maintaining a high index of suspicion, independently reviewing radiologic imaging, and inter-team discussion.

## Introduction

Cranial nerve palsies result from various etiologies, including ischemic, inflammatory, infiltrative, infectious, and compressive causes. Ischemic causes include microangiopathic causes, such as diabetic and hypertensive vasculopathy, giant cell arteritis, and macrovascular events such as a midbrain infarct. Inflammatory causes consist of vasculitis and systemic lupus erythematosus. Infectious causes are widespread, including viral, syphilis, Lyme disease, tuberculosis, and cavernous sinus infections. Lastly, compressive causes are trauma, masses, and aneurysms [1-3].

Third cranial nerve palsies (CN III), or oculomotor nerve palsies, are common cranial nerve palsies. Patients typically present with ptosis, diplopia from exotropia and limited ocular motility, and unilateral mydriasis presenting as anisocoria. The exotropia results from paralysis of the superior rectus, inferior rectus, medial rectus, and inferior oblique muscles. CN III palsies can be acute or chronic, with acute-onset oculomotor palsies requiring urgent, in-depth neurologic and ophthalmologic evaluation to detect the underlying cause [4]. Pupillary involvement is typical in a complete third nerve palsy; however, not all cases present with this finding. One study noted pupillary involvement in 17% of patients with microvascular third nerve palsies and 64% of patients with compressive third nerve palsies; there is an increased likelihood of pupillary involvement in a compressive CN III palsy, but it is not an absolute requirement [5]. Although the incidence of aneurysmal rupture is as low as 1% over the period of one year, this is the most concerning etiology of a CN III palsy [6].

Multiple studies have demonstrated that aneurysms constitute about 10-30% of CN III palsies [3,7,8]. The posterior communicating artery (PCOM) is one of the most common locations for an aneurysm causing CN III palsies. It is associated with a risk of rupture of around 2.5% for aneurysms that are less than 7 mm in size [9]. The most common clinical presentation of a PCOM aneurysm is a CN III palsy [10].

One case report from the Journal of Neuro-Ophthalmology reported four patients with CN III palsies and associated giant cell arteritis (GCA). They concluded that GCA can mimic a microvascular cause of CN III palsies, and patients over 50 years old with an acute third nerve palsy and GCA symptoms should be treated with high-dose steroids and a temporal artery biopsy [11]. Giant cell arteritis is an inflammatory autoimmune condition that causes swelling of the lining of arteries. The condition typically affects patients over 50 years old and has a higher prevalence in women.

We report a case that demonstrates the diagnostic challenges of ophthalmological disease in a medically complex patient and highlights the crucial role of ophthalmologists and inter-team communications. In a patient with a CN III palsy and no other identifiable causes, a thorough, independent review of radiographic imaging should be conducted to rule out an intracranial aneurysm as the source. In addition, reviewing radiologic imaging with the highest qualified radiologists is essential when suspicion for pathology is high.

## Case presentation

Initial presentation

A 64-year-old female presented to the Emergency Department (ED) with a 2-week onset of recurrent worsening diplopia and ptosis of the right eye with an associated right temporal headache and jaw claudication. Her exam showed a visual acuity of 20/70 in the right eye and 20/30 in the left eye; pupils were 4 (dark) → 3 (light) on the right and 3 (dark) → 2 (light) on the left. Her extraocular movements were full bilaterally with significant exotropia on the right. The rest of the anterior segment exam and fundus exam were unremarkable. Her exam was consistent with an incomplete CN III palsy without pupillary involvement. She was immediately admitted to the hospital for further workup.

First admission

Her lab workup showed mildly elevated RBC, hemoglobin, and mildly decreased platelets (Table 1). Her metabolic activities, including thyroid function labs, were normal (Table 2). Her ESR was elevated at 48 mm/hr (Normal 0-20 mm/hr), and CRP was elevated at 1.1 mg/dL (normal 0-0.5 mg/dL) (Table 3). Her myasthenia gravis panel was negative (Table 4). Her urine drug screening was positive for barbiturate, cannabinoid, and cocaine (Table 5).

**Table 1:** Complete blood count WBC = white blood cell, RBC = red blood cell, HGB = hemoglobin

	Parameters	Reference Range
WBC	7.1*10^9^/L	3.9 - 11.7 10^9^/L
RBC	5.33*10^12^/L	3.85 - 5.16 10^12^/L
HGB	16.9 g/dL	12.0 - 15.0 g/dL
Platelet	122*10^9^/L	172 - 440*10^9^/L

**Table 2:** Metabolic activities HbA1c = hemoglobin A1c, TSH = thyroid stimulating hormone, T4 = thyroxine

	Parameters	Reference Range
HbA1c	5.50%	<5.7%
TSH	1.1 uIU/mL	0.35 - 4.94 uIU/mL
T4	0.7 ng/dL	0.7 - 1.5 ng/dL

**Table 3:** Inflammatory markers ESR = erythrocyte sedimentation rate, CRP = C-reactive protein

	Value	Reference Range
ESR	48 mm/hr	0 - 20 mm/hr
CRP	1.1 mg/dL	0 - 0.5 mg/dL

**Table 4:** Myasthenia gravis panel

	Value	Reference Range
Acetylcholine Binding Antibody	0.0 nmol/L	0.0 - 0.4 nmol/L
Acetylcholine Blocking Antibody	23%	0-26%
Muscle-Specific Kinase (MuSK) Antibody	<1:10	<1:10

**Table 5:** Urine drug screen

	Value	Reference Range
Amphetamine Screen, Urine	Negative	Negative
Barbiturate Screen, Urine	Presumptive Positive	Negative
Benzodiazepine Screen, Urine	Negativ	Negative
Cannabinoid Screen, Urine	Presumptive Positive	Negative
Cocaine Screen, Urine	Presumptive Positive	Negative
Fentanyl Screen, Urine	Negative	Negative
Opiate Screen, Urine	Negative	Negative
Phencyclidine (PCP) Screen, Urine	Negative	Negative

CT angiogram read by a tele-radiology service stated no obvious proximal cerebral artery occlusions, mild stenosis of the cavernous carotid arteries, and no report of an aneurysm. MRI read by a tele-radiology service stated chronic basal ganglia lacunar infarcts without a mass or aneurysms. At this time, the differential included GCA (given the symptoms of headache and jaw claudication, mildly elevated ESR, and CRP) aneurysms (but the readings on the angiography were negative), microvascular infarct (given the lack of pupillary involvement in the first presentation and the medical history of diabetes and hypertension), myasthenia gravis, and thyroid orbitopathy (however, the lab workups were negative). Much lower on the differential were an orbital mass, infectious etiologies, and cocaine-associated microangiopathy. Given her complex medical history and pertinent negative workups, she was ultimately treated for presumed GCA with Solumedrol 1000 mg x three days and discharged on a prednisone taper. She underwent a temporal artery biopsy that showed no significant inflammation, giant cells, or histopathologic abnormalities. She was admitted for 10 days for this workup and treatment and was discharged.

Outpatient follow-up

During the next three weeks, she continued her outpatient prednisolone taper, and her symptoms improved during the prolonged prednisone taper but recurred shortly after completing the taper, one month after her initial presentation. She did not seek any medical attention at this time and did not come to her outpatient follow-up.

Second admission

**Figure 1:**
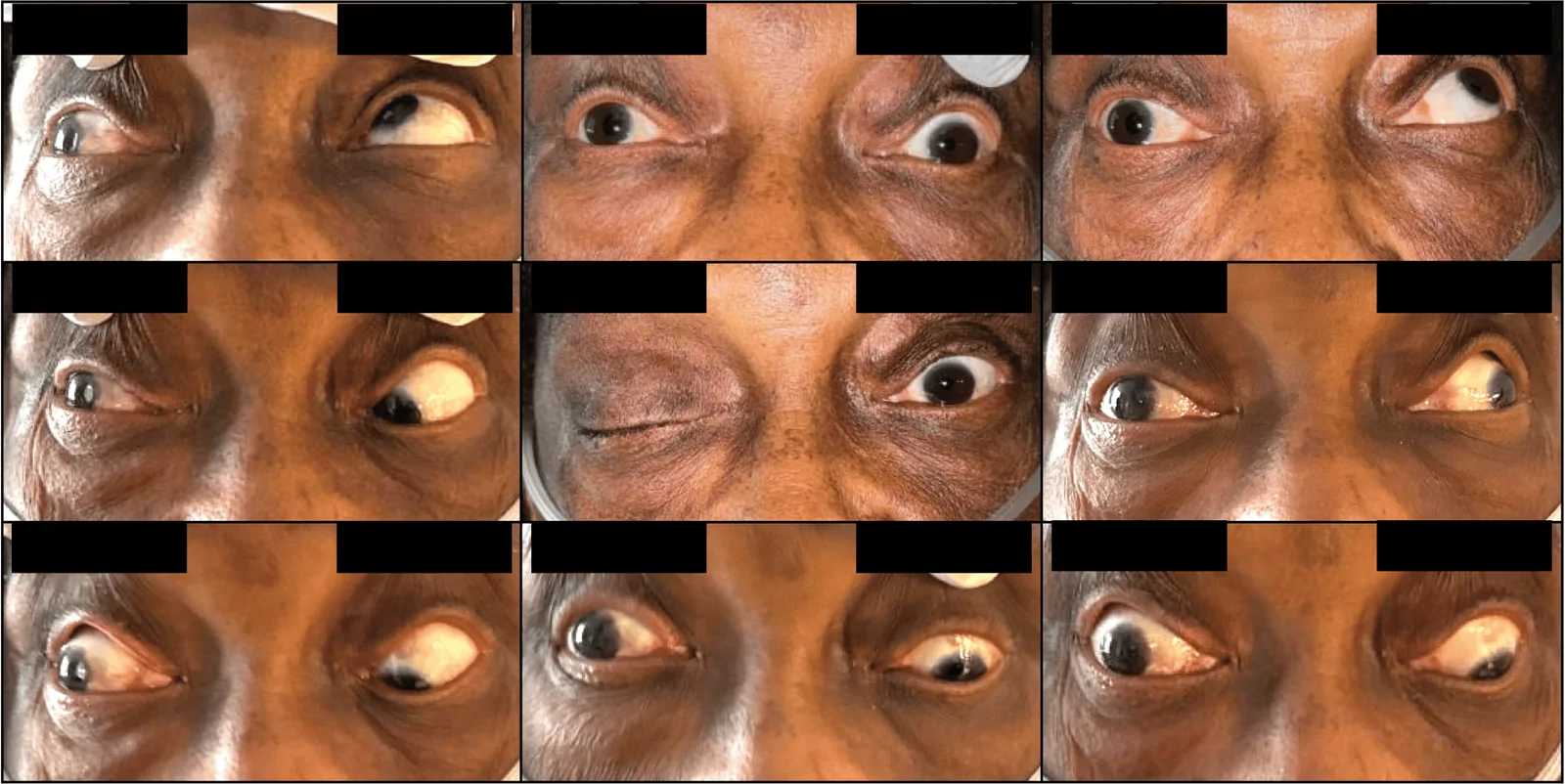
Nine-gaze photograph of the patient with CN III palsy Nine-gaze photograph showing the patient's eye movements in primary gaze and eight directions.

Three months after initial presentation, she returned to the ED for worsening and recurrent symptoms of diplopia and new complete ptosis; she did not have vision changes. Her pupils were 3mm on the right in the dark and the light and 1 mm on the left in the dark and the light. She had right exotropia and hypertropia in primary gaze, and her extraocular movements showed severe restriction to supraduction, adduction, and infraduction consistent with a complete CN III palsy (Figure 1). She was admitted again for additional workup.

**Figure 2:**
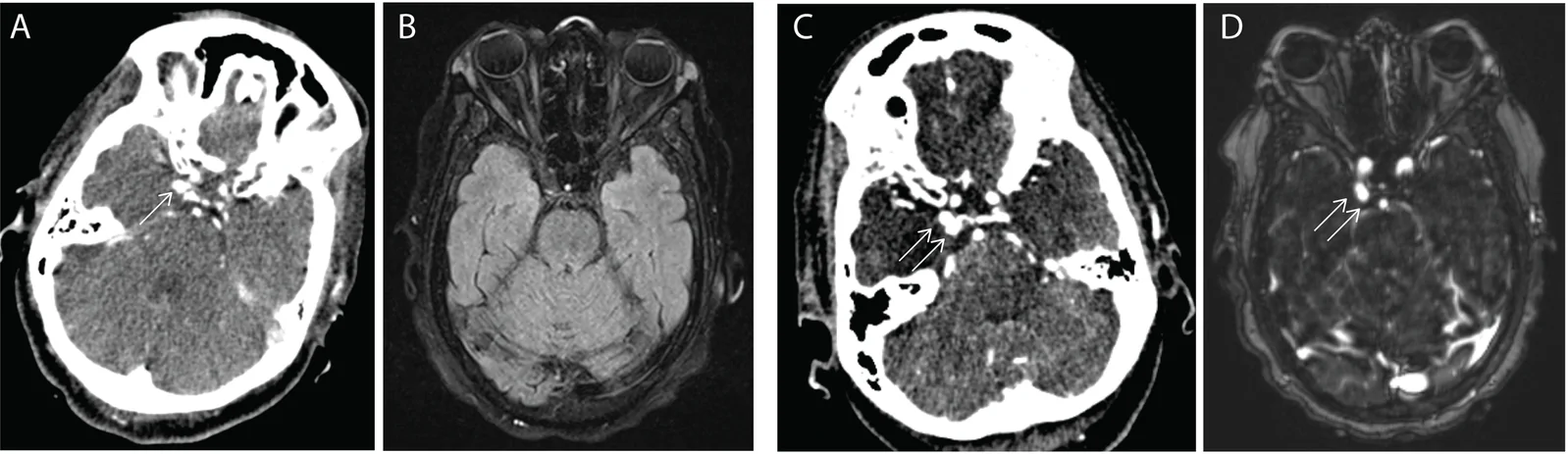
Computed tomography (CT) angiography and MRI angiography showing aneurysms at the posterior communicating artery Axial view of: A) CT angiography at the first admission showing aneurysms (denoted by arrows) at the junction of the posterior communicating artery and the internal carotid artery, and B) MRI brain and orbit at the first admission showing the brain and orbit without any mass lesion. C) Repeat CT angiography and D) MRI angiography at the second admission showing bilobed aneurysms (denoted by arrows) at the junction of the posterior communicating artery and the internal carotid artery.

**Figure 3:**
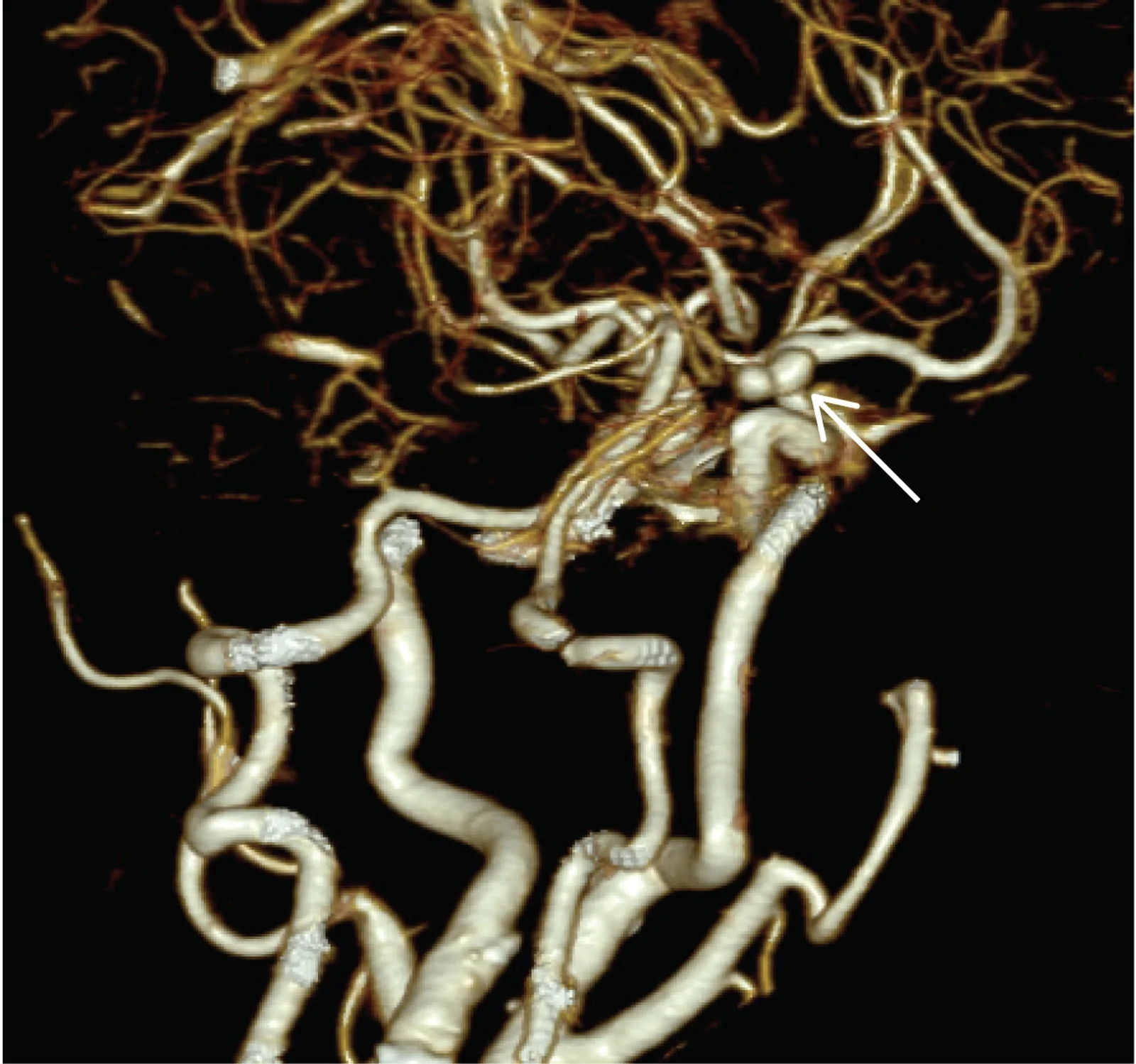
Three-dimensional reconstruction of CT angiography showing an aneurysm at the junction of the posterior communicating artery and the internal carotid artery.

Her dilated fundus exam again had no evidence of dilated vessels or optic disc edema. Repeat CT angiogram (Figure 2A) and MR angiogram (Figure 2B) were both read by radiology as negative, with no aneurysm noted. After the Ophthalmology team reviewed her CT angiogram with neuro-radiology, a contrast-enhancing mass was seen at the junction of the ICA and the PCOM artery (Figures 2C, 2D) that was not on the prior radiology reports. Three-dimensional reconstruction of the CT angiogram was done to highlight the bilobed aneurysm (Figure 3).

**Figure 4:**
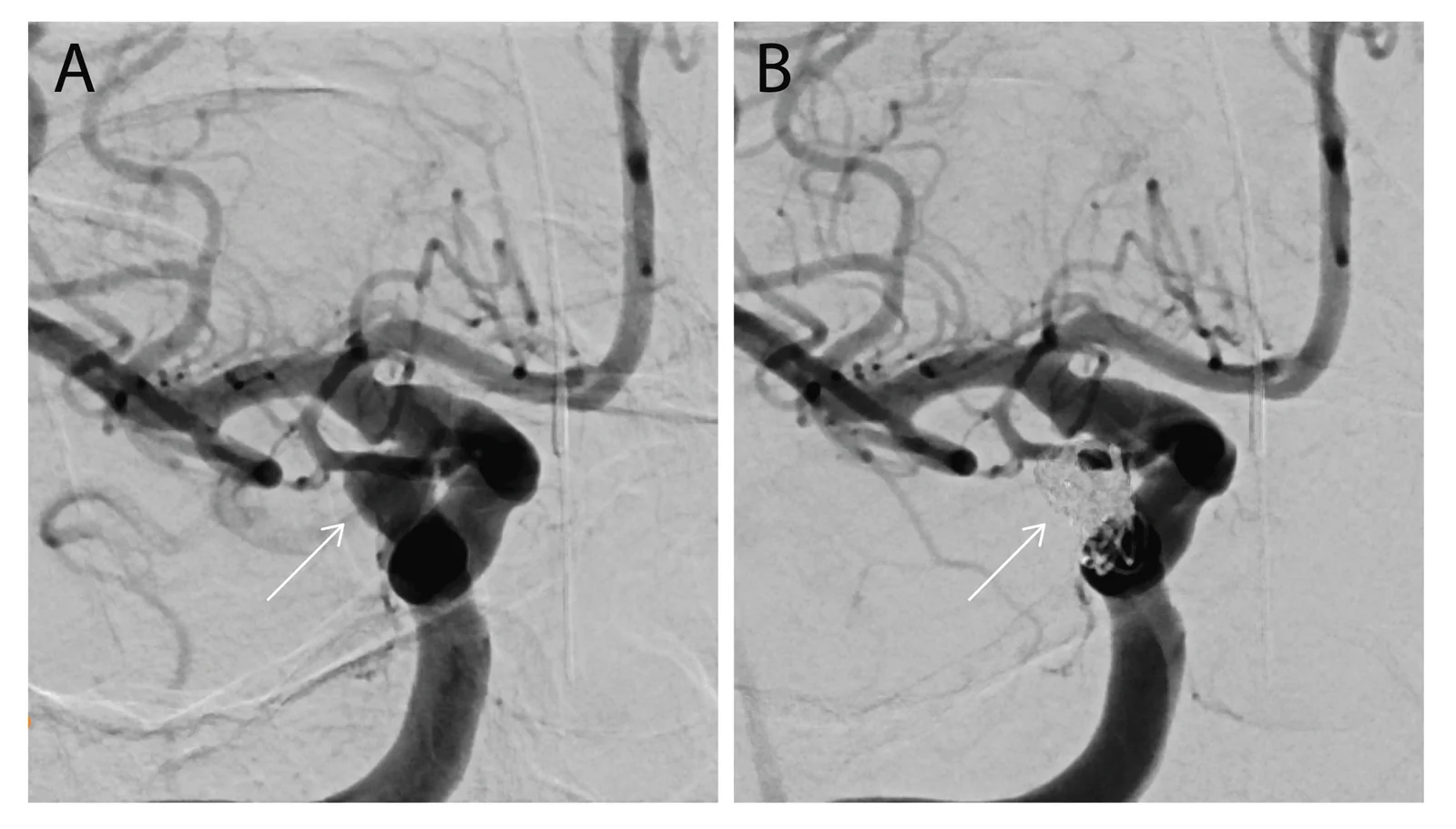
Percutaneous angiography and coil embolism procedure of the aneurysm (denoted by the arrow) A) Aneurysm before coil embolization, and B) Regressed aneurysm without IV contrast fill after the coil embolization The patient was admitted for a total of seven days for the workup and coil embolization before she was discharged.

On independent review of angiographies by ophthalmology and neuroradiology, an 8 mm bilobed aneurysm was found at the right supraclinoid internal carotid artery adjacent to the posterior communicating artery origin, posteriorly directed. The patient was taken to the operating room emergently by neurosurgery for a transcatheter transarterial angiography (Figure 4A) and coil embolization of the aneurysm. The right femoral artery was accessed to reach up to the aortic arch, then the right internal carotid artery. 3D rotational angiography was performed, and an irregular bilobed aneurysm measuring 8.3x4.8 mm projecting posteriorly and laterally from the internal carotid artery and the posterior communicating segment was observed. A step-wise obliteration of the aneurysm was observed with five coils deployed inside the aneurysm, resulting in no filling of either lobe. All surrounding vessels were preserved without coil protrusions. The patient was transported back to the inpatient floor in stable condition (Figure 4B).

Outpatient follow-up

The patient was scheduled for a one-month outpatient follow-up and a six-month repeat angiography, and she did not show up for either appointment. During a phone visit three months later, the patient self-reported that her eyes spontaneously opened and her double vision resolved.

## Discussion

A PCOM aneurysm should be suspected in patients presenting with complete oculomotor nerve (CN III) palsy. A study of 616 patients with an aneurysm causing CN III palsy identified the PCOM artery as the cause in 64.9% of cases [10]. Other causes included the terminal internal carotid artery (29.7%), proximal middle cerebral artery (2.7%), and the basilar tip (2.7%) [10]. Additionally, aneurysms are among the most common causes of CN III palsies according to two studies evaluating different etiologies. In one study of 121 patients in China, intracranial aneurysms (29.8%), diabetic peripheral neuropathy (26.5%), painful ophthalmoplegia (9.9%), trauma (5.8 %), pituitary lesions (5.0%), cavernous sinus disease (5.0%), brainstem infarction (2.5%), brainstem encephalitis (0.8%), and other unknown causes (14.9%) were identified as the causes of CN III palsy [8]. In a second study of 633 patients in Korea, the causes of CN III palsy were identified as microvascular (26.5%), vascular anomalies (17.4%), neoplastic (13.6%), inflammatory (12.5%), miscellaneous (12.2%), idiopathic (9.5%), and traumatic (8.4%) [8].

This case was initially treated as GCA given her symptoms of headache, jaw claudication, elevated ESR and CRP, and negative imaging. Additionally, the initial findings of an incomplete CN III palsy, with no pupillary involvement, weakened the suspicion of an aneurysm as a possible cause of the oculomotor nerve palsy. During the patient’s second visit to the ED, the pupillary involvement prompted a re-evaluation of the radiographs by the ophthalmology team, which was then reviewed by neuro-radiology. Lab work, including a myasthenia gravis panel and drug screen, was ordered to rule out other causes of CN III palsies. The patient’s urine drug screen was positive for cocaine during both admissions. Two separate case studies report pupil-sparing CN III palsy/paresis after cocaine use [12,13]. Both reports stated that the patients presented with diplopia, ptosis, and a headache after cocaine use, similarly to our patient. Additionally, in both reports, the CN III palsy was pupil-sparing [12,13]. While this indicates our patient’s cocaine use could have contributed to her initial presentation of incomplete CN III palsy, the second admission for pupil-involving complete CN III palsy makes this etiology less likely.

Our study highlights the importance of a thorough radiologic evaluation of CT or MRI angiograms in patients with complete CN III palsy. The ophthalmologist should independently review the angiograms and consult the highest qualified radiologists to rule out an aneurysm when suspicion for pathology is high. In one study on the use of artificial intelligence in the detection of unruptured aneurysms, 27.8% of cerebral aneurysms were missed on clinical radiology reports [14]. Additionally, in a study assessing teleradiology medical malpractice cases, deaths of patients occurred more frequently (35%), and a final diagnosis of cerebrovascular disease (9.6%) was more common in teleradiology cases as compared to in-house radiology cases [15]. 

This case demonstrates the importance of interdisciplinary communication. Multiple sub-specialties were consulted to evaluate this patient, including ophthalmology, neurology, rheumatology, and neurosurgery. Each specialty was able to provide important insights that contributed to the ultimate diagnosis and management of this patient’s case. The clinical suspicion for GCA was low, per the rheumatology team, which should prompt further evaluation for other causes of CN III palsy. The patient’s initial improvement in symptoms could have been due to decreased inflammation from the steroid taper; however, this ultimately led to a delay in diagnosis and treatment. Additionally, the initial presentation of incomplete CN III palsy without pupillary involvement, negative initial imaging reports, as well as a complicated social and medical history, all contributed to the diagnostic challenge and delay in treatment for this patient. One limitation of this case report is the limited follow-up and objective examination after the patient’s procedure. The patient was only contacted over the phone to discuss symptoms, and the ophthalmology team was unable to examine the patient to confirm objective improvement. Diagnostic errors are common when patients present with non-classic symptoms, demonstrating the need for a comprehensive review of signs and symptoms across multiple specialties.

## Conclusions

PCOM aneurysms may present as a CN III palsy that may be missed on initial presentation. PCOM aneurysms hold the potential risk of rupture, necessitating prompt identification and early intervention. This case demonstrates the importance of independently reviewing radiologic imaging. Clinicians should suspect an aneurysm, particularly when symptoms recur or when patients do not respond to initial therapy, even if initial imaging is read as negative. In addition, inter-specialty communication is essential when evaluating cases to ensure no critical findings are previously overlooked.
